# ﻿Bryophyte flora of Mount Tebu Forest Reserve, Terengganu, Peninsular Malaysia

**DOI:** 10.3897/phytokeys.234.105783

**Published:** 2023-10-04

**Authors:** Nur Saidatul Atiqah, Elizabeth Pesiu, Muhammad Syafiq Sarimi, Nor Aishah Shafie, Chin Wen Koid, Nik Norhazrina, Nur Syazwana, Gaik Ee Lee

**Affiliations:** 1 Faculty of Science and Marine Environment, 21030 Kuala Nerus, Universiti Malaysia Terengganu, Terengganu, Malaysia; 2 Department of Biological Sciences and Biotechnology, Faculty of Science and Technology, Universiti Kebangsaan Malaysia, 43600 Selangor, Malaysia; 3 Institute of Tropical Biodiversity and Sustainable Development, 21030 Kuala Nerus, Universiti Malaysia Terengganu, Terengganu, Malaysia

**Keywords:** Biodiversity, bryophytes, checklist, Malaysia, Marchantiophyta, taxonomy

## Abstract

A checklist of the bryophyte flora of Mount Tebu Forest Reserve in Terengganu, Peninsular Malaysia, is presented. A total of 189 taxa in 71 genera and 26 families were enumerated. This figure represents 63% of the 298 bryophyte species recorded so far for the State of Terengganu. Out of 189 taxa of bryophytes, 26 liverworts are new additions to the bryoflora of Terengganu. The most prominent liverwort family is represented by Lejeuneaceae, with 54 species from 17 genera, while the moss family is the Sematophyllaceae, with 34 taxa in 13 genera. The majority of the species are epiphytes, either corticolous or ramicolous. Almost half of the bryophyte species have wider elevational ranges and occur from the lowlands to the summit of Mount Tebu.

## ﻿Introduction

**Figure 1.**
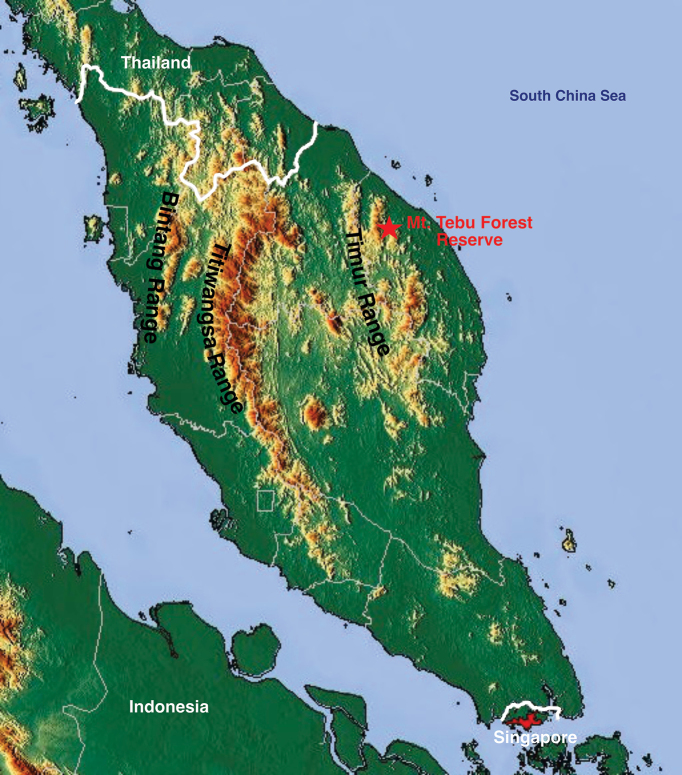
The map of Peninsular Malaysia shows the study area, Mt. Tebu Forest Reserve. Map modified from Dr Blofeld - http://www.maps-for-free.com, CC BY 3.0.

Mount Tebu (1039 m) is the second-highest mountain after Mount Lawit (1519 m) in the northernmost part of Terengganu (Fig. 1). It is located within one of the primary mountain ranges of Peninsular Malaysia, known as the Timur Range (Banjaran Timur). The mountain comprises undulating lowlands, hill and upper hill dipterocarp forest. It has been gazetted as one of the state forest reserves, including the lowlands of Lata Belatan Recreational Forest at the base of Mount Tebu. Geologically, Mount Tebu is composed of unconsolidated alluvium, metasedimentary and igneous rocks in the lowlands to the summit of the mountain (Mohamed and Ali 2014). The unique landscape feature provides ample habitat for a diverse flora and fauna community with high conservation value (see Abdul Rahim et al. (2014) for several extensive floristic and ecological studies). It also offers a variety of vegetation and habitats favourable to the growth and diversity of bryophytes. The history of bryophyte exploration in Terengganu has been reviewed by Lee et al. (2019). The early investigation was conducted by British and Japanese bryologists and yielded only a few bryophyte species, nine being mosses and two were liverworts (Dixon 1926; Yamada 1979; Inoue 1984). Subsequently, more recent collections of bryophytes from this region have been carried out, of which 11 species of bryophyte have been reported for the first time in Peninsular Malaysia and 77 taxa are new records to Terengganu (Lee et al. 2018, 2022; Pesiu et al. 2021; Sarimi et al. 2021).

### ﻿Study area

Mount Tebu Forest Reserve is located at latitude 5.5914°N and longitude 102.6122°E in the Besut District, the northern part of Terengganu. The highest peak reaches 1039 m above sea level, including Lata Belatan Recreational Forest at its base, an entering point to the forest reserve. The foot of this mountain is often shaded by riparian forests where bryophytes are easily found within this area, ranging from 40–100 m a.s.l. with medium canopy cover. The closest rivers are Sungai Besut, Sungai Keluang Besar and Sungai Setiu. Most trees are from the families Dipterocarpaceae, Euphorbiaceae, Annonaceae, Lauraceae and Myrtaceae. They grow on both sides of a valley and throughout the trails. Streams are moderate to fast water currents, often creating a few natural pools on the granite surfaces.

## ﻿Materials and methods

This study is based on the authors’ intensive bryophyte explorations from April 2019–November 2021 in Terengganu and a re-examination of previous moss collections of A. Damanhuri was made during the Mount Tebu scientific expedition in 2012. All the bryophyte samples were collected from various microhabitats along the trails within the study area, including tree trunks and branches, rocks, soils, fallen logs, rotten wood and leaves. Liverwort specimens were deposited in the Herbarium of Universiti Malaysia Terengganu (**UMTP**) and moss specimens were deposited in the Herbarium of Universiti Kebangsaan Malaysia (**UKMB**). About 1000 samples of bryophytes were collected from the study area and were examined by light microscopy. The drawing of the specimen was produced using an Olympus BX43 microscope, equipped with a drawing tube.

## ﻿Results and discussion

**Figure 2.**
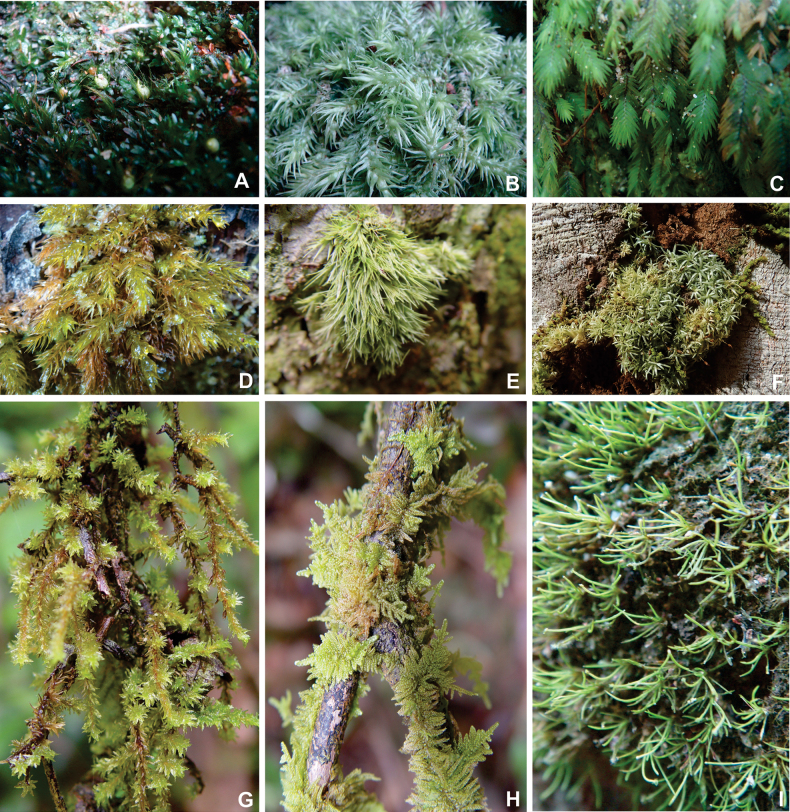
Mosses and their habit **A***Diphysciummucronifolium* Mitt **B***Leucobryumsanctum* (Schwägr.) Hampe **C***Fissidensceylonensis* Dozy & Molk **D***Pyrrhobryumlatifolium* (Bosch & Sande Lac.) Mitt **E***Arthrocormusschimperi* (Dozy & Molk.) Dozy & Molk **F***Octoblepharumalbidum* Hedw **G***Mitthyridiumfasciculatum* (Hook. & Grev.) H. Rob **H***Ectropotheciumbuitenzorgii* (Bél.) Mitt. **I***Syrrhopodonmuelleri* (Dozy & Molk.) Sande Lac.

**Figure 3.**
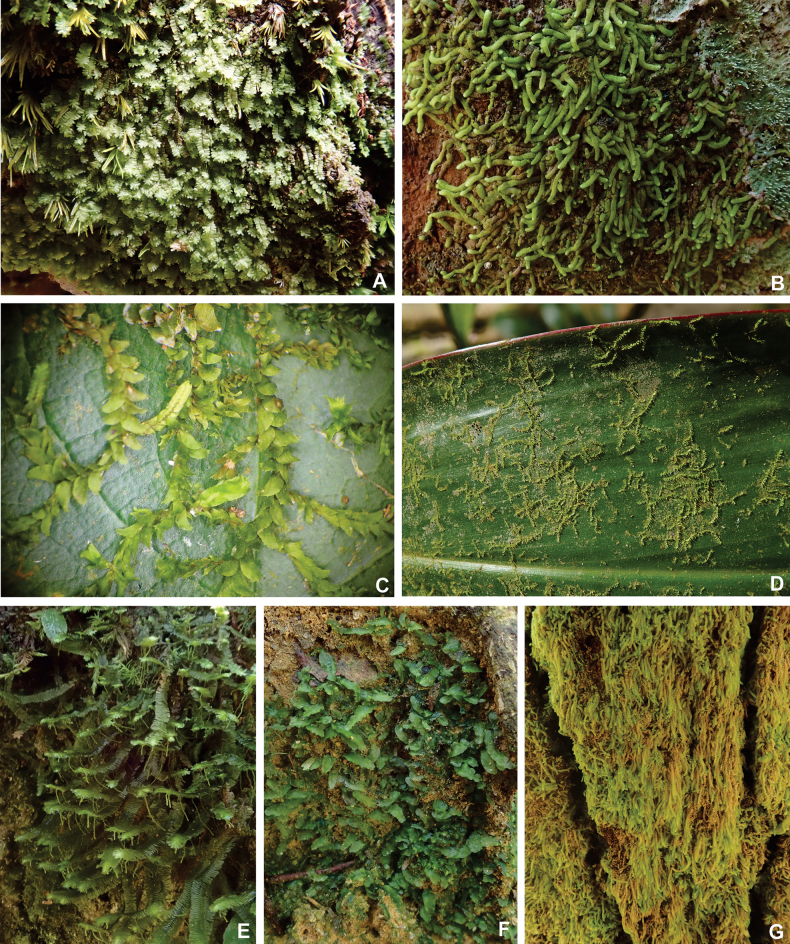
Liverworts and their habit **A***Bazzaniauncigera* (Reinw., Blume & Nees) Trevis **B***Pycnolejeuneagrandiocellata* Steph **C***Caudalejeuneareniloba* (Gottsche) Steph **D***Leptolejeuneaepiphylla* (Mitt.) Steph **E***Bazzaniadensa* (Sande Lac.) Schiffn **F***Pallavicinialyellii* (Hook.) Gray **G***Drepanolejeuneapentadactyla* (Mont.) Steph.

**Figure 4.**
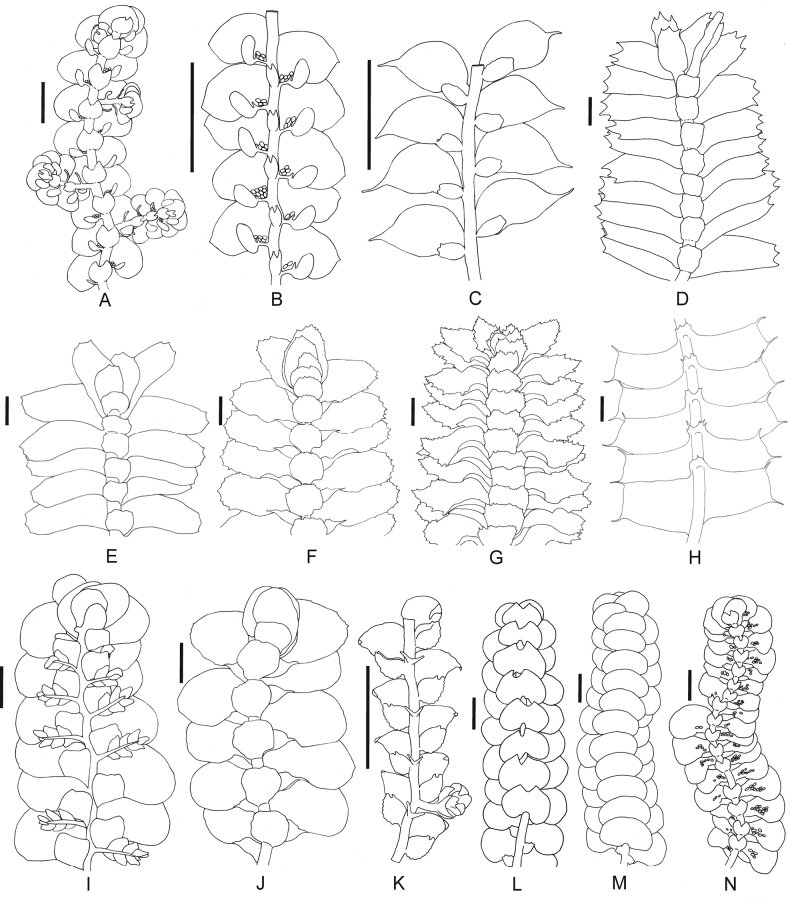
Liverworts from Mount Tebu Forest Reserve, all in ventral view **A***Frullaniagracilis* (Reinw. et al.) Nees **B***Frullaniatrichodes* Mitt **C.***Cololejeuneawightii* Steph **D***Bazzanialongicaulis* (Sande Lac.) Schiffn **E***Bazzaniaalbifolia* Horik **F***Ptychanthusstriatus* (Lehm. & Lindenb.) Nees **G***Thysananthusspathulistipus* (Reinw. et al.) Lindenb **H***Heteroscyphuscoalitus* (Hook.) Schiffn **I***Radulaformosa* (Spreng.) Nees **J***Spruceanthuspolymorphus* (Sande Lac.) Verd **K***Drepanolejeuneavesiculosa* (Mitt.) Steph **L***Lejeuneasordida* (Nees) Nees **M***Lepidolejeuneaintegristipula* (J.B. Jack & Steph.) R.M. Schust **N***Pycnolejeuneagrandiocellata* Steph. (Scale = 0.5 mm).

A total of 189 taxa in 71 genera and 26 families were found in the Mount Tebu Forest Reserve, of which 109 are mosses and 80 are liverworts (Figs 2–4). This represents 63% of the 298 bryophyte species recorded so far for the State of Terengganu (Pócs and Lee 2016; Pesiu et al. 2021; Sarimi et al. 2021; Lee et al. 2022). Out of 80 species of liverworts, 26 are reported for the first time for Terengganu. The largest liverwort family found is the Lejeuneaceae, with 54 species, followed by Lepidoziaceae (eight species) and Radulaceae (seven species). The largest moss family is the Sematophyllaceae, with 34 taxa, followed by Calymperaceae (32 taxa) and Hypnaceae (seven taxa). The smallest liverwort and moss families were represented by only one species, for example, liverworts: Calypogeiaceae, Pallaviciniaceae, Plagiochilaceae, Solenostomataceae and Schistochilaceae and mosses: Diphysciaceae, Myuriaceae, Neckeraceae and Thuidiaceae. As expected, the distinct dominance of species is from the family Lejeuneaceae and mosses Sematophyllaceae and Calymperaceae, representing about 60% of all the bryophyte species found in Mount Tebu. They are the most common bryophyte families in the lowland tropical rainforests with high light intensity, dense canopy, high temperatures and many evergreen tree species.

**Figure 5.**
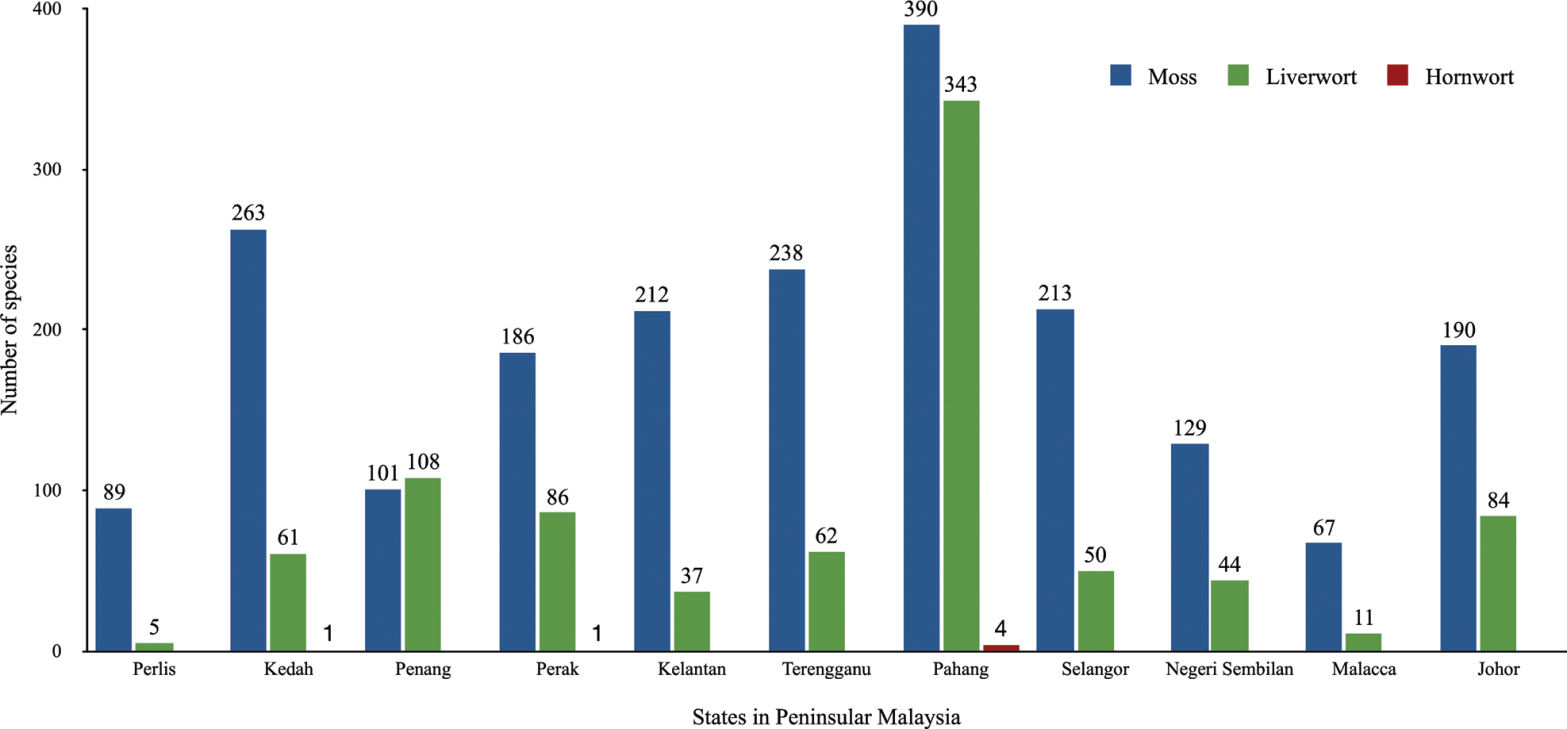
The number of bryophyte species reported from the States of Peninsular Malaysia.

Our study found that the diversity of moss species was higher than that of liverworts, a scenario similar to all the states in Peninsular Malaysia (Fig. 5). Reasons may be lower liverwort collecting, difficulty identifying liverwort species and lack of comprehensive field guides and local bryologists dealing with liverwort. The moss flora of Peninsular Malaysia has been well-studied taxonomically, in which exploration and species inventory of mosses have been more intensive and detailed. Thus far, 524 moss species have been reported from Peninsular Malaysia and all but Perlis and Malacca are well-represented with above 100 species (Yong et al. 2013; Ellis et al. 2019a, b). In comparison, only 491 taxa of liverworts are known from Peninsular Malaysia, suggesting that several States, particularly the northern regions, such as Perlis, Kedah and the east coast (Kelantan), have been under-collected and understudied (Lee and Gradstein 2021; Lee et al. 2022). The State of Pahang seems to be the centre of bryophyte diversity in Peninsular Malaysia (Fig. 5). The presence of major highlands and montane forests in Pahang often provides more favourable and more varied microhabitats for a rich bryophyte flora.

**Figure 6.**
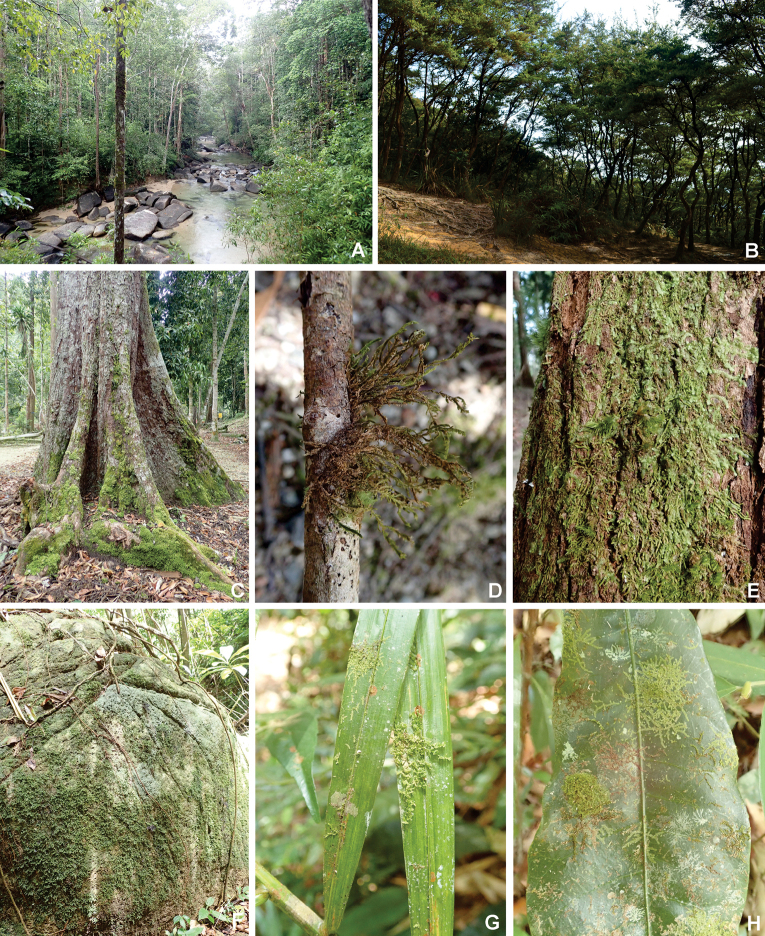
Habitats of bryophyte species of Mount Tebu Forest Reserve **A** lowland dipterocarp forest **B** area around the summit **C–E** bryophytes on tree bases, branches, trunks **F** on rocks **G, H** on leaves.

Most of the bryophyte species in Mount Tebu are epiphytic, growing on the bark of tree trunks, on branches or tree stumps and the base of trees (Fig. 6). About half (49%) of ca. 1000 specimens examined were collected on trees (trunks, branches, twigs), while 22% were from leaves, 14% from rocks, 9% from soil or humus and 6% from rotten logs. About 18 species had broad substrate preferences and occurred on bark and branches of trees, leaves, soils and decaying logs. Others had more narrow preferences and occurred on only one substrate type, for example, *Pallavicinialyellii* and *Solenostomacomatum* were always found on soil, *Ephemeropsistjibodensis* and *Leptolejeuneaepiphylla* occurred exclusively on leaves and *Diphysciummucronifolium* grew only on rock (Appendix 1).

**Figure 7.**
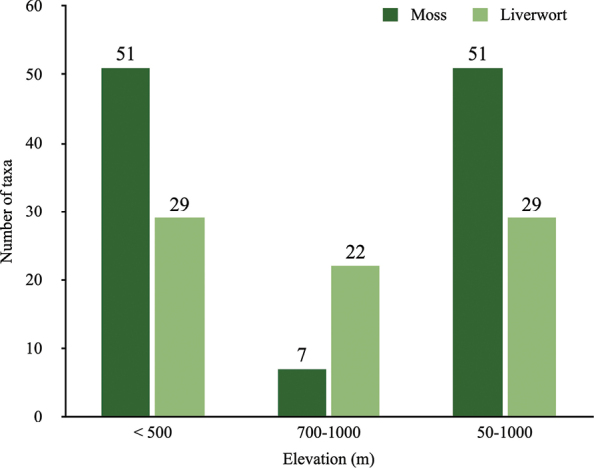
The elevational distribution of bryophyte taxa found in Mount Tebu Forest Reserve.

The distribution of the bryophyte species in Mount Tebu shows a distinct elevational differentiation from sea level to the mountain’s summit (Fig. 7). About half of the moss species have wide elevational ranges and occur from the lowlands to the summit of Mount Tebu. The remaining half of the species have more narrow elevational ranges and are restricted to a lower range, below 500 m. Liverwort species have wider elevational ranges and occur in all elevation belts. However, both groups show a similar trend where most of the species are elevational generalist species, occurring in most rainforest belts and lowland specialists, being found only below 500 m. Of 189 taxa, only 29 species are restricted to the submontane rainforest and occur exclusively at 700–1000 m a.s.l. For example, *Acroporiumcondensatum* and *Mastopomauncinifolium* are obligate highland species known only from Cameron Highlands, Mount Jerai and Mount Tebu (this study) (Tixier 1980; Yong et al. 2006). Other moss species typical of high elevations found in Mount Tebu are *Campylopusexasperatus*, *Leucolomamolle*, Pogonatumcirratumsubsp.macrophyllum, *Acroporiumstramineum* and *Trichosteleumsaproxylophilum* and liverworts are *Frullaniagracilis*, *F.trichodes*, *Cheilolejeuneaceylanica*, *C.trifaria*, *Cololejeuneaaequabilis*, *C.appressa*, *C.equialbi*, *C.falcata*, *C.inflectens*, *C.metzgeriopsis*, *C.obliqua*, *C.ocelloides*, *C.sigmoidea*, *C.stephanii*, *Drepanolejeuneadactylophora*, *Ptychanthusstriatus*, *Schistochilaaligera*, *Spruceanthuspolymorphus* and *Tuyamaellamolischii*.

## ﻿Additional information

### Author ORCIDs

Elizabeth Pesiu https://orcid.org/0000-0003-4063-3489

Nik Norhazrina https://orcid.org/0000-0001-9068-8192

Gaik Ee Lee https://orcid.org/0000-0001-5161-6196

### Data availability

All of the data that support the findings of this study are available in the main text.

## ﻿Appendix 1

**Table A1.** Substrate preferences and elevational distributions of bryophyte taxa in Mount Tebu. Corti: Corticolous (tree trunk), Epi: Epiphyllous (leaf), Ligni: Lignicolous (rotten log), Rami: Ramicolous (tree branch), Saxi: Saxicolous (rock), Terri: Terricolous (soil). An asterisk indicates new additions to the State of Terengganu (*).

No.	Taxon	Substrate preference	Elevation (m)
**Bryophyta (Mosses)**			
**I.**	**Calymperaceae** Kindb.		
1	*Arthrocormusschimperi* (Dozy & Molk.) Dozy & Molk.	Corti, Saxi	60–110
2	*Calymperesafzelii* Sw.	Corti	50–130
3	*Calymperesboulayi* Besch.	Corti	60 –110
4	*Calympereserosum* Müll. Hal.	Corti, Saxi	50–1005
5	*Calymperesfasciculatum* Dozy & Molk.	Corti, Rami	50–1005
6	*Calymperesgraeffeanum* Müll. Hall.	Corti	60–110
7	*Calympereslonchophyllum* Schwägr.	Corti, Ligni	50–970
8	CalympereslonchophyllumSchwägr.subsp.beccari (Hampe) M.Menzel	Ligni	110–940
9	*Calymperesmollucense* Schwägr.	Corti, Rami, Saxi, Ligni	50–130
10	*Calymperesporrectum* Mitt.	Corti	60–110
11	*Exostratumblumii* (Hampe) L.T.Ellis	Corti, Saxi, Ligni	60–110
12	*Leucophanesaugustifolium* Renauld & Cardot	Corti, Saxi, Terri	50–1005
13	*Leucophanesglaucum* (Schwägr.) Mitt.	Corti	50–130
14	*Leucophanesoctoblepharioides* Brid.	Corti, Rami, Saxi	50–970
15	*Mitthyridiumconstrictum* (Sull.) H.Rob	Corti, Epi, Rami	60–110
16	Mitthyridiumfasciculatumsubsp.cardotii (M.Fleisch.) B.C.Tan & L.T.Ellis	Corti	50–130
17	*Mitthyridiumfasciculatum* (Hook. & Grev.) H.Rob.	Corti, Rami	50–1005
18	*Mitthyridiumflavum* (Müll. Hal.) H.Rob.	Corti	50–130
19	*Mitthyridiumjunquilianum* (Mitt.) H.Rob.	Corti, Rami	50–130
20	*Mitthyridiumrepens* (Harv.) H.Rob.	Corti	50–970
21	*Mitthyridiumundulatum* (Dozy & Molk.) H.Rob.	Corti, Rami	50–970
22	*Octoblepharumalbidum* Hedw.	Corti	50–130
23	*Syrrhopodonalbo-vaginatus* Schwägr.	Ligni	50–130
24	*Syrrhopodonaristifolius* Mitt.	Corti	50–1005
25	*Syrrhopodonconfertus* Sande Lac.	Corti	50–1005
26	*Syrrhopodoncroceus* Mitt.	Corti, Saxi	50–1005
27	*Syrrhopodonmuelleri* (Dozy & Molk.) Sande Lac.	Corti	50–970
28	*Syrrhopodonprolifer* Schwägr.	Corti, Ligni	110–940
29	*Syrrhopodonspiculosus* Hook. & Grev.	Corti, Ligni	50–970
30	*Syrrhopodonstoneae* W.D.Reese	Corti	50–130
31	*Syrrhopodontrachyphyllus* Mont.	Corti	50–940
32	*Syrrhopodontristichus* Schwägr.	Corti	60–940
**II.**	**Daltoniaceae** Schimp.		
33	*Distichophyllumcuspidatum* (Dozy & Molk.) Dozy & Molk.	Corti	110–940
34	Distichophyllumnigricaulevar.cirratum (Renauld & Cardot) M.Fleisch	Corti, Saxi	110–940
35	*Ephemeropsistjibodensis* K.I.Goebel	Epi	60–110
**III.**	**Dicranaceae** Schimp.		
36	*Campylopusericoides* (Griff.) A.Jaeger	Saxi	100–1005
37	*Campylopusexasperatus* (Nees & Blume) Brid.	Saxi	940–1005
38	*Dicranellacoarctata* (Müll. Hal.) Bosch & Sande Lac.	Terri	110–940
39	*Leucolomaamoene-virens* Mitt.	Saxi	50–130
10	*Leucolomamolle* (Müll. Hal.) Mitt.	Corti	940–1005
**IV.**	**Diphysciaceae** M.Fleisch		
41	*Diphysciummucronifolium* Mitt.	Saxi	60–970
**V.**	**Fissidentaceae** Schimp.		
42	*Fissidensceylonensis* Dozy & Molk.	Saxi	60–110
43	*Fissidenscrassinervis* Sande Lac.	Terri	50–970
44	*Fissidenshollianus* Dozy & Molk.	Corti	60–110
45	*Fissidensjavanicus* Dozy & Molk.	Saxi	60–110
46	*Fissidensoblongifolius* Hook. f. & Wilson	Corti	70–80
47	*Fissidenspellucidus* Hornsch.	Terri	70–80
**VI.**	**Hypnaceae** Schimp.		
48	*Ectropotheciumbuitenzorgii* (Bél.) Mitt.	Corti, Ligni, Saxi, Terri	50–970
49	*Ectropotheciumichnotocladum* (Müll. Hal.) A.Jaeger	Corti, Saxi	50–940
50	*Isopterygiumalbescens* (Hook. in Schwägr.) A.Jaeger	Corti	50–130
51	*Pseudotaxiphyllumpohliaecarpum* (Sull. & Lesq.) Z.Iwats.	Terri	110–940
52	*Vesiculariadubyana* (Müll. Hal.) Broth.	Corti, Saxi, Terri	50–130
53	*Vesiculariamiquelii* (Sande Lac.) M.Fleisch.	Corti	60–110
54	*Vesiculariareticulata* (Dozy & Molk.) Broth.	Saxi	70–80
**VII.**	**Hypnodendraceae** Broth.		
55	*Hypnodendrondendroides* (Brid.) Touw	Saxi	110–940
56	Hypnodendronsubspininervium(Müll. Hal.)A.Jaegersubsp.arborescens (Mitt.) Touw	Corti	110–940
**VIII.**	**Leucobryaceae** Schimp.		
57	*Leucobryumaduncum* Dozy & Molk.	Corti, Ligni, Saxi, Terri	50–970
58	Leucobryumaduncumvar.scalare (M.Fleisch.) A.Eddy	Corti	50–970
59	*Leucobryumbowringii* Mitt.	Corti, Saxi	50–970
60	*Leucobryumcandidum* (P.Beauv.) Wilson	Corti, Saxi	50–970
61	*Leucobryumchlorophyllosum* Müll. Hal.	Corti	50–970
62	*Leucobryumjavense* (Brit.) Mitt.	Corti, Terri	100–1005
63	*Leucobryummicroleucophanoides* A.Johnson	Corti	100–970
64	*Leucobryumsanctum* (Schwägr.) Hampe	Corti, Saxi, Terri	50–970
**IX.**	**Meteoriaceae** Kindb.		
65	*Aerobryidiumcrispifolium* (Broth. & Geh.) M.Fleisch.	Corti, Rami	60–110
66	*Aerobryopsislongissima* (Dozy & Molk.) M.Fleisch.	Corti	50–130
**X.**	**Myuriaceae** M.Fleisch.		
67	*Oedicladiumpseudorufescens* (Hampe) B.C.Tan & Mohamed	Corti, Saxi	50–970
**XI.**	**Neckeraceae** Schimp.		
68	*Himantocladiumplumula* (Nees in Brid.) M.Fleisch.	Corti	60–110
**XII.**	**Polytrichaceae** Schwägr.		
69	Pogonatumcirratumsubsp.fuscatum (Mitt.) Hyvönen	Terri	110–940
70	Pogonatumcirratumsubsp.macrophyllum (Dozy & Molk.) Hyvönen	Saxi	940–1005
**XIII.**	**Pottiaceae** Hampe		
71	*Barbulaconsanguinea* (Thwaites & Mitt.) A.Jaeger	Saxi	60–110
72	*Hyophilainvoluta* (Hook.) A.Jaeger	Saxi, Terri	50–130
**XIV.**	**Rhizogoniaceae** Broth.		
73	*Pyrrhobryumlatifolium* (Bosch & Sande Lac.) Mitt.	Corti	50–130
74	*Pyrrhobryummedium* (Besch.) Manuel	Corti	60–110
**XV.**	**Sematophyllaceae** Broth.		
75	*Acanthorrhynchiumpapillatum* (Harv.) M.Fleisch.	Corti, Ligni	50–130
76	*Acroporiumadspersum* (Hampe) Broth.	Corti	60–110
77	*Acroporiumcondensatum* E.B.Bartram	Saxi	940–1005
78	*Acroporiumdiminutum* (Brid.) M.Fleisch.	Corti	60–1005
79	*Acroporiumjoannis-winkleri* Broth.	Corti, Ligni, Terri	60–1005
80	*Acroporiumlamprophyllum* Mitt.	Corti	50–130
81	*Acroporiumrigens* (Dixon) Dixon	Saxi, Terri	50–1005
82	*Acroporiumstramineum* (Reinw. & Hornsch.) M.Fleisch.	Terri	940–1005
83	*Acroporiumstrepsiphyllum* (Mont.) B.C.Tan	Corti, Saxi	60–1005
84	*Clastobryophilumbogoricum* (Bosch & Sande Lac.) M.Fleisch.	Corti	50–130
85	*Clastobryumcaudatum* (Sande Lac.) M.Fleisch.	Corti	70–80
86	*Clastobryumcuculligerum* (Sande Lac.) Tixier	Corti	60–110
87	*Clastobryumepiphyllum* (Renauld & Cardot) B.C.Tan & Touw	Corti, Rami	60–110
88	*Gammiellatonkinensis* (Broth. & Paris) B.C.Tan	Rami	100–970
89	*Isocladiellasurcularis* (Dixon) B.C.Tan & Mohamed	Corti	60–110
90	*Mastopomauncinifolium* (Broth.) Broth.	Rami	940–1005
91	*Meiotheciummicrocarpum* (Harv.) Mitt.	Corti	50–130
92	*Papillidiopsisbruchii* (Dozy & Molk.) W.R.Buck & B.C.Tan	Corti	60–110
93	*Papillidiopsiscomplanata* (Dixon) W.R.Buck & B.C.Tan	Corti, Ligni	50–1005
94	*Papillidiopsisluxurians* (Dozy & Molk.) W.R.Buck & B.C.Tan	Corti, Ligni, Saxi	50–940
95	*Papillidiopsismalesiana* W.R.Buck & B.C.Tan	Corti	50–130
96	*Rhaphidostichumbunodicarpum* (Müll. Hal.) M.Fleisch.	Corti, Saxi	50–130
97	*Rhaphidostichumpiliferum* (Broth.) Broth.	Corti	60–110
98	*Taxitheliuminstratum* (Brid.) Broth.	Corti	50–130
99	*Taxitheliumisocladium* (Bosch & Sande Lac.) Renauld & Cardot	Corti, Epi, Rami	50–130
100	*Taxitheliumkerianum* (Broth.) Broth.	Corti, Rami	60–940
101	*Taxitheliumlindbergii* (A.Jaeger) Renauld & Cardot	Epi, Rami	110–1005
102	*Taxitheliumnepalense* (Schwägr.) Broth.	Corti	60–110
103	*Trichosteleumboschii* (Dozy & Molk.) A.Jaeger	Corti, Ligni, Rami, Saxi	50–1005
104	*Trichosteleumsaproxylophilum* (Müll. Hal.) B.C.Tan *et al*.	Terri	940–1005
105	*Trichosteleumsingapurense* M.Fleisch.	Corti	70-80
106	*Trichosteleumstigmosum* Mitt.	Corti, Ligni, Rami	50–130
107	*Trismegistialancifolia* (Harv.) Broth.	Corti, Saxi	50–970
108	Trismegistialancifoliavar.pseudoplicata (Harv.) Broth.	Corti, Ligni	60–940
**XVI.**	**Thuidiaceae** Schimp.		
109	*Thuidiumpristocalyx* (Müll. Hal.) A.Jaeger	Saxi	50–940
	**Marchantiophyta (Liverworts)**		
**I.**	**Calypogeiaceae** Arnell		
1	**Asperifoliaarguta* (Nees & Mont.) A.V.Troitsky et al.	Terri	63–340
**II.**	**Frullaniaceae** Lorch		
2	*Frullaniaapiculata* (Reinw. *et al*.) Nees	Epi	850–1000
3	**Frullaniagracilis* (Reinw. *et al*.) Nees	Corti	980
4	**Frullaniatrichodes* Mitt.	Epi	800
**III.**	**Lejeuneaceae** Cavers		
5	*Caudalejeuneareniloba* (Gottsche) Steph.	Corti, Epi, Rami	40–1039
6	*Ceratolejeuneaminor* Mizut.	Epi	100
7	*Ceratolejeuneasingapurensis* (Lindenb.) Schiffn.	Epi	100
8	*Cheilolejeuneaceylanica* (Gottsche) R.M.Schust. & Kachroo	Corti, Epi	900–1000
9	*Cheilolejeuneatrapezia* (Nees) Kachroo & R.M.Schust.	Corti, Epi, Rami	80–1000
10	*Cheilolejeuneatrifaria* (Reinw. *et al*.) Mizut.	Epi	1006
11	*Cololejeuneaaequabilis* (Sande Lac.) Schiffn.	Epi	900–1000
12	*Cololejeuneaappressa* (A.Evans) Benedix	Epi	600–1000
13	*Cololejeuneaequialbi* Tixier	Epi	880–1000
14	*Cololejeuneafalcata* (Horik.) Benedix	Epi	600–1000
15	*Cololejeuneafloccosa* (Lehm. & Lindenb.) Schiffn.	Epi	80–1000
16	*Cololejeuneainflata* Steph.	Epi	80–1000
17	*Cololejeuneainflectens* (Mitt.) Benedix	Epi	900–1000
18	*Cololejeunealanciloba* Steph.	Epi	80–500
19	*Cololejeuneametzgeriopsis* (K.I.Goebel) Gradst. et al.	Epi	780
20	*Cololejeuneaobliqua* (Nees & Mont.) Schiffn.	Epi	800–1000
21	*Cololejeuneaocelloides* (Horik.) Mizut.	Epi	900–1000
22	*Cololejeuneaplanissima* (Mitt.) Abeyw.	Epi	80–1000
23	*Cololejeuneaschmidtii* Steph.	Epi	300–1000
24	*Cololejeuneasigmoidea* Jovet-Ast & Tixier	Epi	800–1000
25	*Cololejeuneastephanii* Benedix	Epi	900–1006
26	*Cololejeuneaverrucosa* Steph.	Epi	100–900
27	**Cololejeuneawightii* Steph.	Corti	100–900
28	*Coluraacroloba* (Prantl) Jovet-Ast	Epi	100–900
29	*Coluraari* (Steph.) Steph.	Epi	100–1000
30	*Coluraconica* (Sande Lac.) K.I.Goebel	Corti, Epi	100–900
31	*Coluracorynophora* (Nees *et al*.) Trevis.	Corti, Epi	100–1000
32	*Colurainuii* Horik.	Epi	100–1000
33	*Drepanolejeuneadactylophora* (Nees *et al*.) J.B.Jack & Steph.	Epi	850–1006
34	*Drepanolejeunealevicornua* Steph.	Epi	80–1000
35	*Drepanolejeunealongicornua* (Herzog) Mizut.	Epi	100–1000
36	*Drepanolejeuneapentadactyla* (Mont.) Steph.	Epi	100–1000
37	*Drepanolejeuneaspicata* (Steph.) Grolle & R.L.Zhu	Corti, Epi, Rami	100–1000
38	*Drepanolejeuneaternatensis* (Gottsche) Schiffn.	Corti, Epi, Rami	100–1000
39	*Drepanolejeuneathwaitesiana* (Mitt.) Steph.	Epi	80–1000
40	**Drepanolejeuneavesiculosa* (Mitt.) Steph.	Epi	60–100
41	*Lejeuneaadpressa* Nees	Corti, Epi	90–500
42	*Lejeuneamicholitzii* Mizut.	Epi	900–1000
43	**Lejeuneasordida* (Nees) Nees	Corti	89
44	*Lepidolejeuneabidentula* (Steph.) R.M.Schust.	Corti, Epi	63–340
45	**Lepidolejeuneaintegristipula* (J.B.Jack & Steph.) R.M.Schust.	Corti	63–340
46	*Leptolejeuneaamphiophthalma* Zwickel	Epi	80–1000
47	*Leptolejeuneasubacuta* A.Evans	Epi	300–1000
48	*Leptolejeuneaepiphylla* (Mitt.) Steph.	Epi	80–1000
49	*Leptolejeuneamaculata* (Mitt.) Schiffn.	Epi	80–1000
50	*Leptolejeuneavitrea* (Nees) Schiffn.	Epi	80–1000
51	*Lopholejeuneaeulopha* (Taylor) Schiffn.	Epi	100–900
52	*Metalejeuneacucullata* (Reinw. *et al*.) Grolle	Epi	900–1000
53	*Microlejeuneapunctiformis* (Taylor) Steph.	Corti, Epi	89–940
54	**Ptychanthusstriatus* (Lehm. & Lindenb.) Nees	Corti	980
55	**Pycnolejeuneagrandiocellata* Steph.	Corti	60–100
56	**Spruceanthuspolymorphus* (Sande Lac.) Verd.	Corti	980
57	*Tuyamaellamolischii* (Schiffn.) S.Hatt.	Epi	780–1006
58	**Thysananthusspathulistipus* (Reinw. *et al*.) Lindenb.	Rami	48
**IV.**	**Lepidoziaceae** Limpr.		
59	**Bazzaniaalbifolia* Horik.	Corti	89–700
60	**Bazzaniaasymmetrica* (Steph.) N.Kitag.	Corti, Rami	100–200
61	**Bazzaniacalcarata* (Sande Lac.) Schiffn.	Corti	100
62	**Bazzaniadensa* (Sande Lac.) Schiffn.	Corti	89–700
63	**Bazzanialongicaulis* (Sande Lac.) Schiffn.	Corti, Terri	89–700
64	**Bazzaniauncigera* (Reinw. *et al*.) Trevis.	Corti, Saxi	89–700
65	**Kurziagonyotricha* (Sande Lac.) Grolle	Terri	89
66	**Lepidoziatrichodes* (Reinw. *et al*.) Nees	Terri	89
**X.**	**Lophocoleaceae** Vanden Berghen		
67	**Heteroscyphusaselliformis* (Reinw. *et al*.) Schiffn.	Corti	89
68	**Heteroscyphuscoalitus* (Hook.) Schiffn.	Terri	89
69	**Heteroscyphussucculentus* (Gottsche) Schiffn.	Corti	89
**XI.**	**Pallaviciniaceae** Mig.		
70	**Pallavicinialyellii* (Hook.) Gray	Terri	60
**XII.**	**Plagiochilaceae** Müll.Frib.		
71	*Plagiochilabantamensis* (Reinw. *et al*.) Mont.	Corti,	89
**XIII.**	**Radulaceae** Müll.Frib.		
72	*Radulaacuminata* Steph.	Epi	80–1000
73	*Radulaassamica* Steph.	Epi	60–100
74	**Radulaformosa* (Spreng.) Nees	Corti	60–100
75	*Radulagrandilobula* Promma & Chantanaorr.	Epi	100
76	*Radulajavanica* Gottsche	Corti, Epi	60–100
77	*Radulanymannii* Steph.	Epi	60–100
78	*Radulatjibodensis* K.I.Goebel	Epi	80–1000
**IX.**	**Solenostomaceae** Stotler & Crand.-Stotl.		
79	**Solenostomacomatum* (Nees) C.Gao	Terri	60
**X.**	**Schistochilaceae** H.Buch		
80	**Schistochilaaligera* (Nees & Blume) J.B.Jack & Steph.	Corti	994
